# Preparation of high temperature NH_3_-SCR catalysts with carbonate as precursors by ball milling method

**DOI:** 10.1039/d2ra06552e

**Published:** 2022-12-08

**Authors:** Na Wang, Lei Wang, Huidong Xie, Yang Liu, Yepeng Sun, Chang Yang, Chengmin Ge

**Affiliations:** College of Architecture and Civil Engineering, Xi'an University of Science and Technology Xi'an 710054 Shaanxi China wangna811221@xust.edu.cn +86-29-82202335 +86-29-82203378; School of Chemistry and Chemical Engineering, Xi'an University of Architecture and Technology Xi'an 710055 Shaanxi China; Engineering Comprehensive Training Center, Xi'an University of Architecture and Technology Xi'an 710055 Shaanxi China; Shandong Dongyuan New Material Technology Co., Ltd. Dongying 257300 Shandong China

## Abstract

High-temperature 10Ce–2La/TiO_2_ catalysts for selective catalytic reduction of NO with NH_3_ were prepared by the ball milling, impregnation and co-precipitation methods and their catalytic performance was compared. The effects of different starting materials of the ball milling method on the catalytic activity were investigated. The results showed that the 10Ce–2La/TiO_2_ catalyst prepared by the ball milling method using carbonates as starting materials exhibited the highest NO conversion, which was more than 80% in the temperature range of 330–550 °C. The as-prepared catalysts were characterized by XRD, TEM, and XPS. Results showed that the ball milling prepared 10Ce–2La/TiO_2_ had the advantages of uniform active site distribution, high oxygen storage capacity, and high Ce^3+^ and O_α_ ratio. The results of NH_3_-TPD and H_2_-TPR showed that the ball milling method not only improved the redox ability but also increased the quantities and concentration of the acidic sites. The green production and economically viable concept of this process provides a new solution for the production application of industrial catalysts.

## Abstract 2

High-temperature 10Ce–2La/TiO_2_ catalysts for selective catalytic reduction of NO with NH_3_ were prepared by the ball milling, impregnation and co-precipitation methods with different precursors and their catalytic performance was compared.

## Graphical abstract

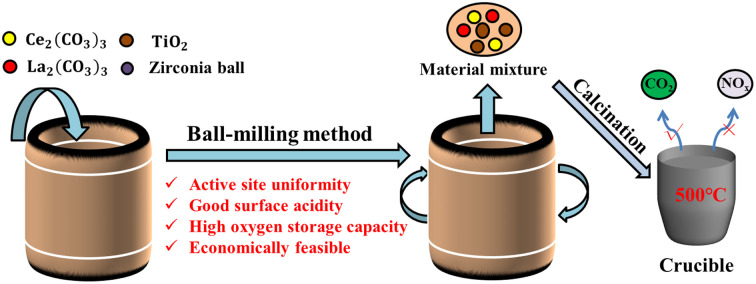

## 1. Introduction

In modern society, the increase in air pollution due to harmful gases released from fossil energy consumption has become a global problem. These harmful gases mainly include NO_*x*_, SO_*x*_, and CO, and so on. Among them, NO_*x*_ mainly originates from the flue gas of stationary sources represented by boilers in coal-fired plants and exhaust from the mobile sources represented by vehicles. The NO_*x*_ emissions cause environmental problems such as acid rain, photochemical smog, ozone layer depletion, and greenhouse effect.^1,2^ Therefore, it is required to study a technique to diminish the NO_*x*_ emission. Ammonia selective catalytic reduction (NH_3_-SCR) technology is currently the most widely used NO removal technology for flue gas. The most used commercial catalysts for NH_3_-SCR are vanadium-based catalysts such as V_2_O_5_–WO_3_ (MoO_3_)/TiO_2_. Although vanadium based catalyst has high NO conversion rate, it also has many disadvantages, such as narrow temperature operation window, high vanadium toxicity and poor high temperature activity.^3,4^ With that regards, non-vanadium-based catalysts have been explored and studied. Among them, cerium oxides have excellent oxidation properties as well as oxygen storage capacity due to the special electron distribution of cerium.^5,6^ At the same time, because cerium oxide has no biological toxicity, it is currently considered to be a powerful substitute for vanadium based catalysts. In our previous report, we prepared a series of Ce–La/TiO_2_ high-temperature NH_3_-SCR catalysts by the impregnation method.^7^ The introduction of La_2_O_3_ improved the catalytic activity and the Ce–La/TiO_2_ catalysts had a wide temperature window (355–590 °C) and better catalytic performance than commercial vanadium-based catalysts at high temperature.

The catalytic activity and N_2_ selectivity of catalysts are influenced not only by the active components, but also by the preparation method and starting materials.^8,9^ In some cases, although the precursor for the preparation of catalysts does not change the final elemental composition of the catalyst, it can have an impact on the catalytic performance of the catalyst. Xu^10^*et al.* prepared Fe–TiO_2_ catalysts *via* a co-precipitation method and found that different TiO_2_ precursors (titanium sulfate, titanium tetrachloride, *n*-butyl titanate and commercial TiO_2_) had a significant effect on the catalytic activity. Among them, the samples synthesized with titanium sulfate as the precursor showed the highest catalytic activity. As for the preparation methods, Chen^11^*et al.* compared the catalytic performance of the Ce–Mn/TiO_2_ mixed oxides prepared by inverse co-precipitation, conventional co-precipitation and impregnation methods. Results showed that the catalyst prepared by inverse co-precipitation method had the highest low-temperature catalytic activity and the best resistance to water and SO_2_. Pan *et al.*^12^ prepared a series of catalysts with different mixing ratios by ball milling. The catalysts prepared by the ball milling methods showed NO conversion more than 90%, good N_2_ selectivity and high toxicity resistance to SO_2_ and H_2_O in a wide temperature range (200–400 °C). It is well known that the ball milling method is an easy industrial production method with the advantages of simple operation, low cost, and easy to scale up. In particular, the ball milling method can reduce the discharge of waste water and harmful exhaust gases when compared with the impregnation and co-precipitation methods that often used in the industrial production, thus contributing to environmental protection.^13,14^ Although the Ce–La/TiO_2_ catalysts prepared by the impregnation method of our group have excellent high-temperature catalytic performance, the raw materials contain a large amount of nitrates, which will exhaust NO_*x*_ during the following calcination. Therefore, if catalysts with comparable performance to that of the impregnation method can be produced by a ball milling method, it will be of great help to save cost and reduce the emission of wastewater and waste gas. To our best knowledge, there are no reports on the preparation of Ce–La/TiO_2_ catalysts by ball milling method using carbonates as the starting materials. Also, there is no report on the comparison of the catalytic activity of the Ce–La/TiO_2_ catalysts prepared by different methods.

In this work, in order to compare the effects of ball milling, impregnation and co-precipitation on the catalytic activity, and to minimize pollution during the catalyst preparation, Ce–La/TiO_2_ catalysts with the same mass ratio were prepared using different methods or precursors (NO_3_^−^, OH^−^, CO_3_^2−^). All catalysts conduct catalytic reaction in the same simulated flue gas environment. In order to analyze the denitration performance of the catalyst from its phase, morphology, specific surface area, acidic sites and redox capacity, a series of characterizations such as XRD, SEM, BET, XPS, H_2_-TPR and NH_3_-TPD were carried out. The apparent activation energy and turn over frequency (TOF) of NH_3_-SCR catalyst were calculated.

## 2. Experimental

### 2.1 Materials and reagents

#### 2.1.1. Qualification of reagents

All chemicals and reagents were purchased without further purification. Ce(NO_3_)_3_.6H_2_O, La(NO_3_)_3_·6H_2_O, NH_3_·H_2_O and citric acid are of analytical grade, and other reagent are of industrial grade.

#### 2.1.2. Synthesis of catalysts

First, cerium carbonate and lanthanum carbonate were weighed and mixed according to Ce_*x*_La_*y*_Ti_88_, where *x* and *y* were the weight ratio of CeO_2_ and La_2_O_3_ to 88 parts of anatase TiO_2_. Then the materials were placed in a nylon jar and ground on a KQM-Z/B planetary ball miller in the presence of appropriate amount of deionized water and zirconia balls. The rotation speed and time were set as 500 rpm and 1 h, respectively. Every 20 min, the rotation direction was changed. After the ball milling, the zirconia balls were separated through a sieve and the slurry was dried at 105 °C for 12 h. Finally, the samples were calcined at 500 °C for 4 h to obtain the catalyst for measurement, denoted as BC-10Ce–2La/TiO_2_. For comparison, 10Ce–2La/TiO_2_ catalysts were prepared by different methods (Table 1). The catalyst prepared by the ball milling method using Ce(OH)_4_ and La(OH)_3_ instead of Ce_2_(CO_3_)_3_ and La_2_(CO_3_)_3_ as raw materials was denoted as BH-10Ce–2La/TiO_2_. The catalysts prepared by the impregnation method^7^ and co-precipitation method^11^ according to the literature were labeled as IN-10Ce–2La/TiO_2_ and CN-10Ce–2La/TiO_2_, respectively.

**Table 1:** List of the starting materials and the ratio of the four different catalysts

Preparation method	Starting materials	Abbreviation
Ball milling	Ce_2_(CO_3_)_3_, La_2_(CO_3_)_3_, TiO_2_	BC-10Ce–2La/TiO_2_
Ball milling	Ce(OH)_4_, La(OH)_3_, TiO_2_	BH-10Ce–2La/TiO_2_
Impregnation	Ce(NO_3_)_3_·6H_2_O, La(NO_3_)_3_, 6H_2_O, TiO_2_	IN-10Ce–2La/TiO_2_
Co-precipitation	Ce(NO_3_)_3_·6H_2_O, La(NO_3_)_3_, 6H_2_O, TiO_2_	CN-10Ce–2La/TiO_2_

### 2.2 Instrumental measurements

An X-ray diffractometer (Thermo ARL SCINTAG X'TRA) was used to examine the phase composition of the samples. The X-ray source was Cu K_α_ radiation (wavelength 0.154056 nm) operated at an accelerating voltage of 40 kV and a tube current of 40 mA. The sample was tested by N_2_ adsorption automatic pore specific surface area analyzer (Builder SSA-7300). The specific surface area (m^2^ g^−1^), pore volume and average pore diameter of the catalyst were calculated from the desorption branch of the isotherm in the pressure range of 0.05–0.30 by Brunauer–Emmett–Teller (BET) equation, *t*-plot method and Barrett–Joyner–Halenda (BJH) model, respectively. The morphology of the catalyst was observed on a transmission electron microscope (TEM, Talos F200x, FEI, USA) with a Super-X energy dispersive spectrometer (EDS) for the test of the elemental mappings. Before the observation, the sample was dispersed in anhydrous ethanol and dried. X-ray photoelectron spectroscopy (XPS) was performed on a Thermo Scientific K-Alpha electron spectrometer with an Al Kα (*hν* = 1486.6 eV) radiation source operated at 12 kV with a vacuum pressure of 3 × 10^−5^ Pa. All binding energies were calibrated by the standard C1s peak (284.8 eV). Temperature programmed desorption/reduction (TPD/TPR) experiments were carried out on a chemisorption apparatus (Micromeritics, AutoChem II 2920). First, appropriate amount of sample was weighed and put into a reaction tube, and then heated from room temperature to 350 °C for surface impurities removal. Then, the sample was purged with He gas flow for 1 h and cooled to 50 °C. For NH_3_-TPD, 10% NH_3_/He mixture was injected for 1 h until the adsorption was saturated, then He gas flow was introduced for 1 h to remove weak physical adsorption of NH_3_ on the surface. Finally, the temperature was raised to 700 °C at a heating rate of 10 °C min^−1^ in He flow and a TCD detector was used for the detection of the NH_3_ desorption. For H_2_-TPR, 10% H_2_/Ar mixture was used and the sample was heated to 1000 °C at a heating rate of 5 °C min^−1^ and detected the reducing gas with a TCD.

### 2.3 Catalytic test

First, the as-prepared catalysts were ground in a KQM-Z/B planetary ball miller and coated to a honeycomb cordierite carrier with size of *φ*20 × *L*50. After drying in an oven, the loaded cylinders were placed in a quartz tube furnace to test the catalytic activity. The loading weight of the catalyst was 1 g. The fed gas mixture consisted of 500 ppm NO, 500 ppm NH_3_, 5% H_2_O, 3% O_2_ and the balanced N_2_. The total gas flow was 2500 mL min^−1^, hence the corresponding gas hourly space velocity (GHSV) was calculated to be 150 000 mL g^−1^ h^−1^. The outlet concentrations of NO and NO_2_ were measured using an ECOM flue gas analyzer (Germany), and the outlet N_2_O concentration was detected using a KRM50 infrared analyzer. The temperature control was performed by a computer program. The NO conversion and N_2_ selectivity were calculated according to the following equations:^15^1
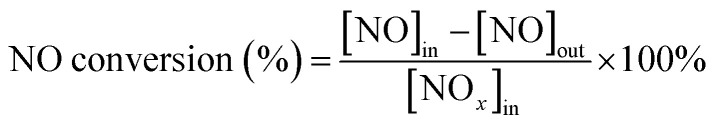
2
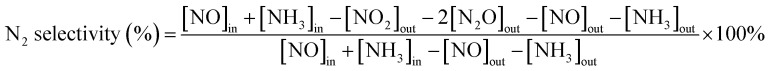
where, [ ]_in_ and [ ]_out_ represent the inlet and outlet concentrations of different gases at steady state, respectively.

At high GHSV, assuming that the NH_3_-SCR catalytic reaction is not controlled by diffusion but by the dynamic mode, the normalized SCR reaction rate constant per specific surface area of the catalyst can be calculated according to the following equation:^16,17^3
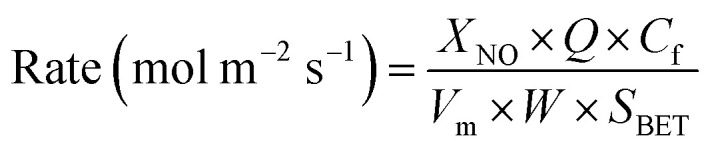
where *X*_NO_ is the NO conversion (%) at different temperatures (250–350 °C with an interval of 25 °C), *Q* is the volumetric flow (2500 mL min^−1^), *C*_f_ is the fed NO concentration (500 ppm), *V*_m_ is the molar volume of gas under standard conditions (22.4 L mol^−1^), *W* is the weight of the catalyst (g), and *S*_BET_ is the specific surface area of the catalyst (m^2^ g^−1^).

The turnover frequency (TOF) values were used to compare the catalytic rates of the different catalysts. To ensure that the SCR reaction was not controlled by diffusion, the NO conversion was controlled below 20% by increasing GHSV and the TOF value of NO at the active center Ce was calculated by the following equation:^18,19^4
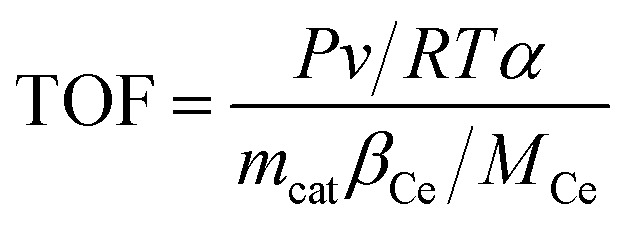
where *P* is the standard atmospheric pressure (1.01 × 10^5^ Pa), *v* is the flow of NO (129 mL min^−1^), *R* is the molar gas constant (8.314 J mol^−1^ K^−1^), *T* is the test temperature: 473, 488, 503, 518, 533 and 543 K, *α* is the NO conversion (%), *m*_cat_ is the weight of the catalyst (1 g), *β*_Ce_ is the loading ratio of Ce (%) calculated using XPS data, and *M*_Ce_ is the molecular weight of cerium (140.1 g mol^−1^).

## 3. Results and discussion

### 3.1 Characterization of catalysts

**Fig. 1:**
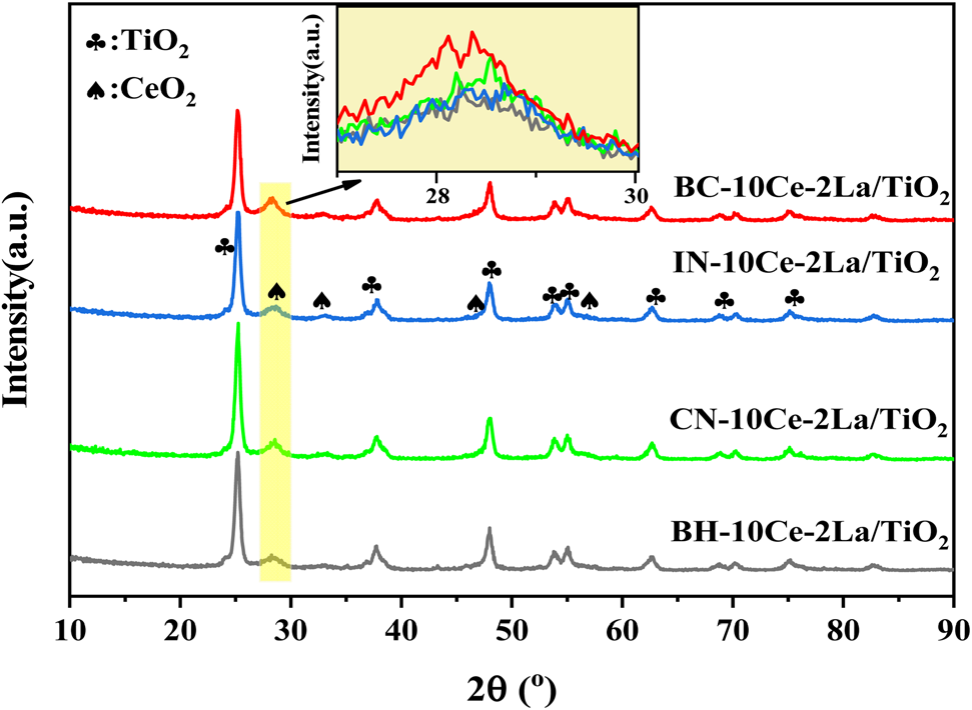
XRD pattern of BH-10Ce–2La/TiO_2_, CN-10Ce–2La/TiO_2_, IN-10Ce-2La/TiO_2_ and BC-10Ce-2La/TiO_2_ catalysts.

Fig. 1 shows the X-ray diffraction patterns of the four catalysts. It can be seen that the main phase of all samples was anatase TiO_2_ (JCPDS no. 21-1272). Weak diffraction peaks of ceriamite CeO_2_ (JCPDS no. 43-1002) were detected near 28.6°, 33°, *etc.* Among them, BC-10Ce–2La/TiO_2_ has the strongest CeO_2_ peak (111 crystal plane), while BH-10Ce–2La/TiO_2_ has the weakest diffraction peak, indicating that the precursors can induce CeO_2_ crystallization to different degree. The high crystallization of BC-10Ce–2La/TiO_2_ catalyst can promote the activation of NH_3_,^3^ which is consistent with its high catalytic activity. No peaks of La_2_O_3_ species were observed for all samples, which might be related to their low content, poor crystallinity and high dispersion on the catalyst surface.^20^

Table 2 lists the specific surface area (*S*_BET_), pore volume (*V*_P_) and average pore size (*D*_P_) of the catalysts measured by N_2_ adsorption and desorption method. As can be seen from the table, except for BH-10Ce–2La/TiO_2_, the surface area and pore volume, pore size of other three catalysts showed almost no difference, which might be due to the same content of TiO_2_ as the carrier. The results showed that the catalysts prepared by the ball milling method with carbonates as the precursors had comparable temperature windows and catalytic activities with the conventional methods.

**Table 2:** Specific surface area, pore volume and average pore size of the catalysts

Samples	BET surface area^a^ (m^2^ g^−1^)	Pore volume^b^ (cm^3^ g^−1^)	Average pore size^c^ (nm)
BC-10Ce–2La/TiO_2_	79	0.043	1.08
IN-10Ce–2La/TiO_2_	80	0.042	1.06
CN-10Ce–2La/TiO_2_	80	0.042	1.05
BH-10Ce–2La/TiO_2_	74	0.040	1.08

a Calculated by BET method.

b Calculated by *t*-plot method.

c Calculated by BJH method.

**Fig. 2:**
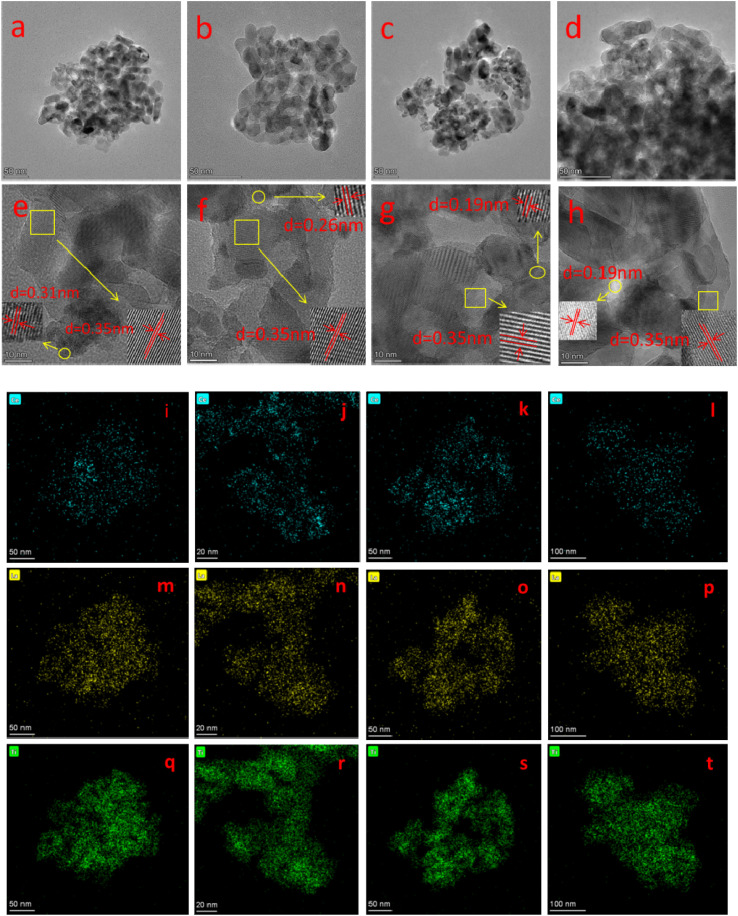
(a–d) TEM image, (e–h) HR-TEM, and (i–l) Ce, (m–p) La, (q–t) Ti mapping of BH-10Ce–2La/TiO_2_, CN-10Ce–2La/TiO_2_, IN-10Ce–2La/TiO_2_, and BC-10Ce–2La/TiO_2_.

Fig. 2 shows the TEM images, HR-TEM images and the elemental mapping of the four catalysts. It can be seen from Fig. 2(a)–(d) that all catalysts have similar morphology and are porous structures formed by the accumulation of nanoparticles of different sizes, which helps to exhibit good catalytic activity.^21^ From the HR-TEM images in Fig. 2(e)–(h), a crystalline spacing of 0.35 nm can be observed in all the catalysts, corresponding to the (101) crystalline plane of anatase TiO_2_.^22^ The *d* spacing of 0.31, 0.26 and 0.19 nm correspond to the (111), (200) and (220) crystalline planes of CeO_2_, respectively.^23^ It is noteworthy that no lattice striations of the crystalline lanthanide oxide can be observed for all samples, suggesting that it exists on the catalyst surface in an amorphous structure.^24^ This is in agreement with the XRD results. Moreover, the elemental mappings of Ce, La and Ti (Fig. 2(i)–(t)) show that Ce, La and Ti elements are uniformly distributed in the catalysts.

**Fig. 3:**
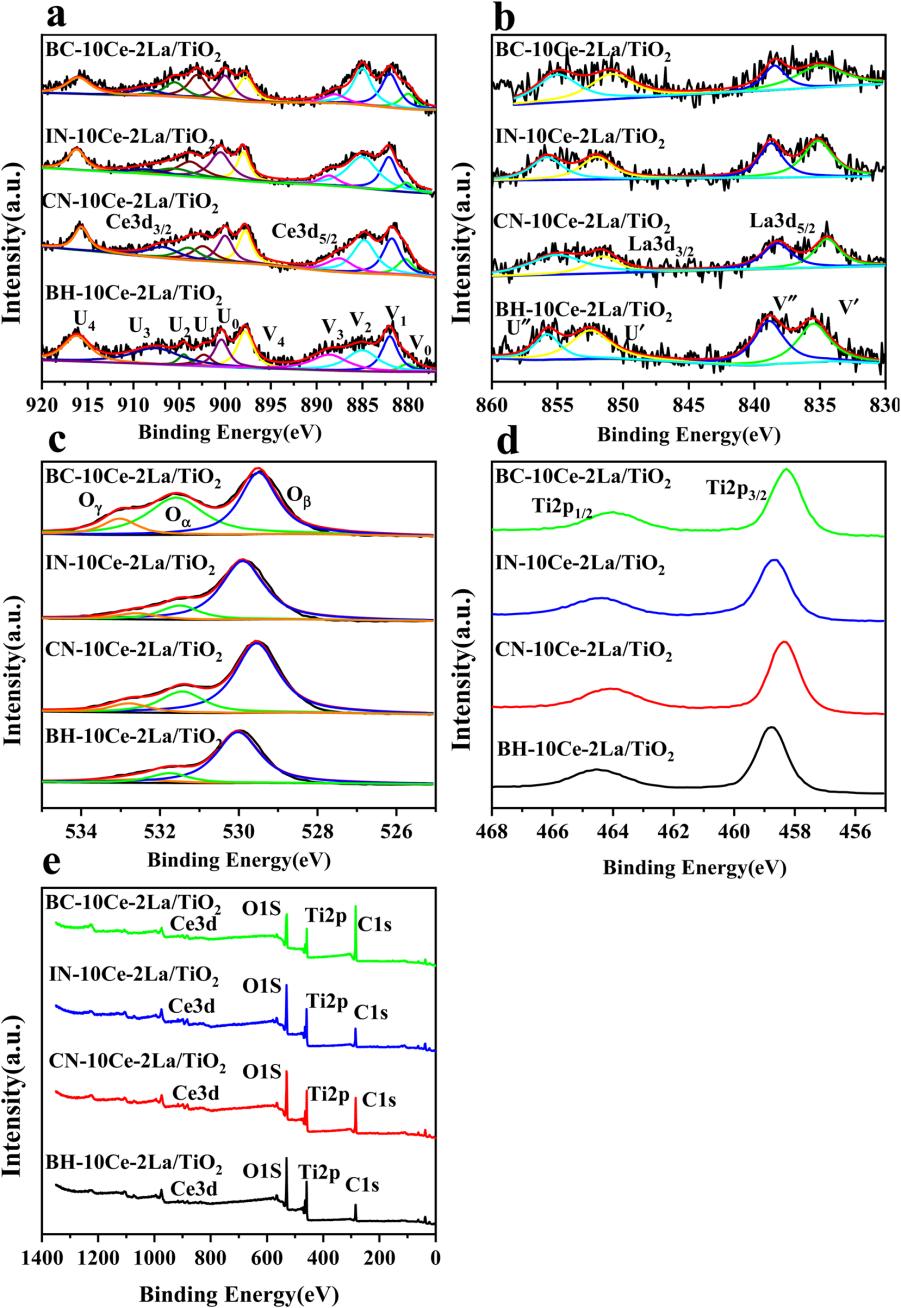
XPS of BH-10Ce–2La/TiO_2_, CN-10Ce–2La/TiO_2_, IN-10Ce–2La/TiO_2_ and BC-10Ce–2La/TiO_2_ catalysts. (a) Ce 3d; (b) La 3d; (c) O 1s; (d) Ti 2p; (e) Survey spectra.

XPS was used to detect the electrovalence and the relative proportions of elements on the surface of the catalysts and shown in Fig. 3. Ten peaks of Ce 3d in Fig. 3(a) can be fitted, and the peaks labeled as “V” and “U” represents the 3d_5/2_ and 3d_3/2_ spin–orbit states of cerium, respectively. The peaks labeled V_1_, V_3_, and V_4_ are from Ce^4+^ 3d_5/2_, while the peaks labeled U_1_, U_3_, and U_4_ represent Ce^4+^ 3d_3/2_. In addition, the peaks of V_0_, V_2_ and U_0_, U_2_ belong to Ce^3+^ 3d_5/2_ and Ce^3+^ 3d_3/2_, respectively. The presence of V_0_/U_0_ and V_2_/U_2_ double peaks indicates that the catalyst contains some oxygen vacancies and is in a partially reduced state.^25^Table 3 lists the surface element concentrations and the ratios of Ce^3+^ and O_α_ for the catalysts calculated by XPS. The relative content of Ce^3+^ in these samples can be calculated by the following equation for the integral peak area ratio of Ce^3+^ and total Ce:^26^ Ce^3+^ (%) = (*S*_V_0__ + *S*_V_2__ + *S*_U_0__ + *S*_U_2__)/∑(*S*_U_ + *S*_V_) × 100% and is presented in Table 3. It can be seen that the highest percentage of Ce^3+^/(Ce^3+^+Ce^4+^) on the surface of IN-10Ce–2La/TiO_2_ catalyst is 48.87%, while those on BC-10Ce–2La/TiO_2_, CN-10Ce–2La/TiO_2_ and BH-10Ce–2La/TiO_2_ are 42.52%, 41.18% and 30.46%, respectively. It is noteworthy that BH-10Ce–2La/TiO_2_ exhibits the lower Ce^3+^ and O_α_ ratios than that of IN-10Ce–2La/TiO_2_ and CN-10Ce–2La/TiO_2_. The low Ce^3+^ ratio of BH-10Ce–2La/TiO_2_ can be attributed to the starting material of Ce(OH)_4_. On the other hand, a higher proportion of Ce^3+^ can promote the generation of oxygen vacancies, unsaturated chemical bonds and charge imbalance on the catalyst surface, thus further promoting the NO oxidation reaction.^27,28^

**Table 3:** Surface element concentrations and ratios of Ce^3+^ and O_α_ calculated by XPS

Catalysts	Surface atomic concentration (atom%)				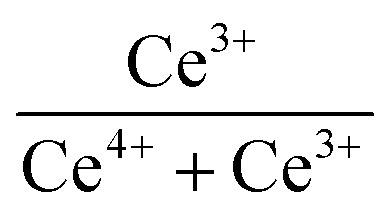	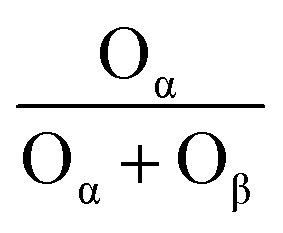
	O	Ce	La	Ti		
BC-10Ce–2La/TiO_2_	76.92	1.99	0.48	20.64	42.52%	47.62%
IN-10Ce–2La/TiO_2_	69.37	3.05	0.57	26.99	48.87%	17.89%
CN-10Ce–2La/TiO_2_	69.19	2.44	0.46	24.24	41.18%	23.85%
BH-10Ce–2La/TiO_2_	69.19	1.36	0.54	27.30	30.46%	17.72%

In Fig. 3(b), both the XPS peaks of La 3d_5/2_ and 3d_3/2_ exhibit double splitting, one is attributed to spin–orbit interactions and the other is electron transfer from the valence band of oxygen to the empty La 4f energy level orbital.^29^ Compared with BC-10Ce–2La/TiO_2_, the peaks of IN-10Ce–2La/TiO_2_ and BH-10Ce–2La/TiO_2_ shifted toward the high binding energy direction, which indicated that the interaction between Ce and La of BC-10Ce–2La/TiO_2_ was enhanced. In Fig. 3(c), the lower binding energy (529.5–530 eV) of O1s is attributed to lattice oxygen, denoted as O_β_; the higher binding energy (531.4–531.8 eV) is attributed to surface unsaturated oxygen, denoted as O_α_; and the highest peak (532–532.8 eV) corresponds to adsorbed water, denoted as O_γ_.^7,10^ Because O_α_ has a higher mobility than the lattice oxygen O_β_, O_α_ can lead to form more surface oxygen vacancies and subsequently be more highly active in oxidation reactions. As can be seen from Table 3, the O_α_ concentration (76.92%) and O_α_/(O_α_ + O_β_) ratio (47.62%) of BC-10Ce–2La/TiO_2_ are the highest among all catalysts, which is favorable for the oxidation of NO to NO_2_ in the “fast SCR” mechanism.^30^ BC-10Ce–2La/TiO_2_ has the second highest Ce^3+^ and the highest O_α_ ratio. The catalytic activity of BC-10Ce–2La/TiO_2_ is slightly greater than that of IN-10Ce–2La/TiO_2_ and CN-10Ce–2La/TiO_2_, indicating that both Ce^3+^ and O_α_ ratio have an effect on the catalytic activity. The reason for the difference in Ce^3+^ and O_α_ ratio may be due to the difference of the new generated CeO_2_ decomposed by Ce_2_(CO_3_)_3_, Ce(NO_3_)_3_, Ce(OH)_3_ (for CN-10Ce–2La/TiO_2_), and Ce(OH)_4_ of the different method. Also, the ball milling method accounts for the difference in Ce^3+^ and O_α_ ratio, because the mechanical movement is benefit to improve the O_α_ ratio.^31^ Therefore, the ball milling process can be used to control the dispersion of the active component on the catalyst surface and improve the redox characteristics by increasing the Ce^3+^ and O_α_ ratio. In the XPS of Ti 2p (Fig. 3(d)), two broad peaks are observed at approximately 458.5 eV and 464.5 eV for all samples, showing Ti presents in the highest oxidation valence Ti^4+^.^30,32^ In addition, all the peaks of Ce, La, O, Ti and C can be found in the survey XPS of Fig. 3(e).

**Fig. 4:**
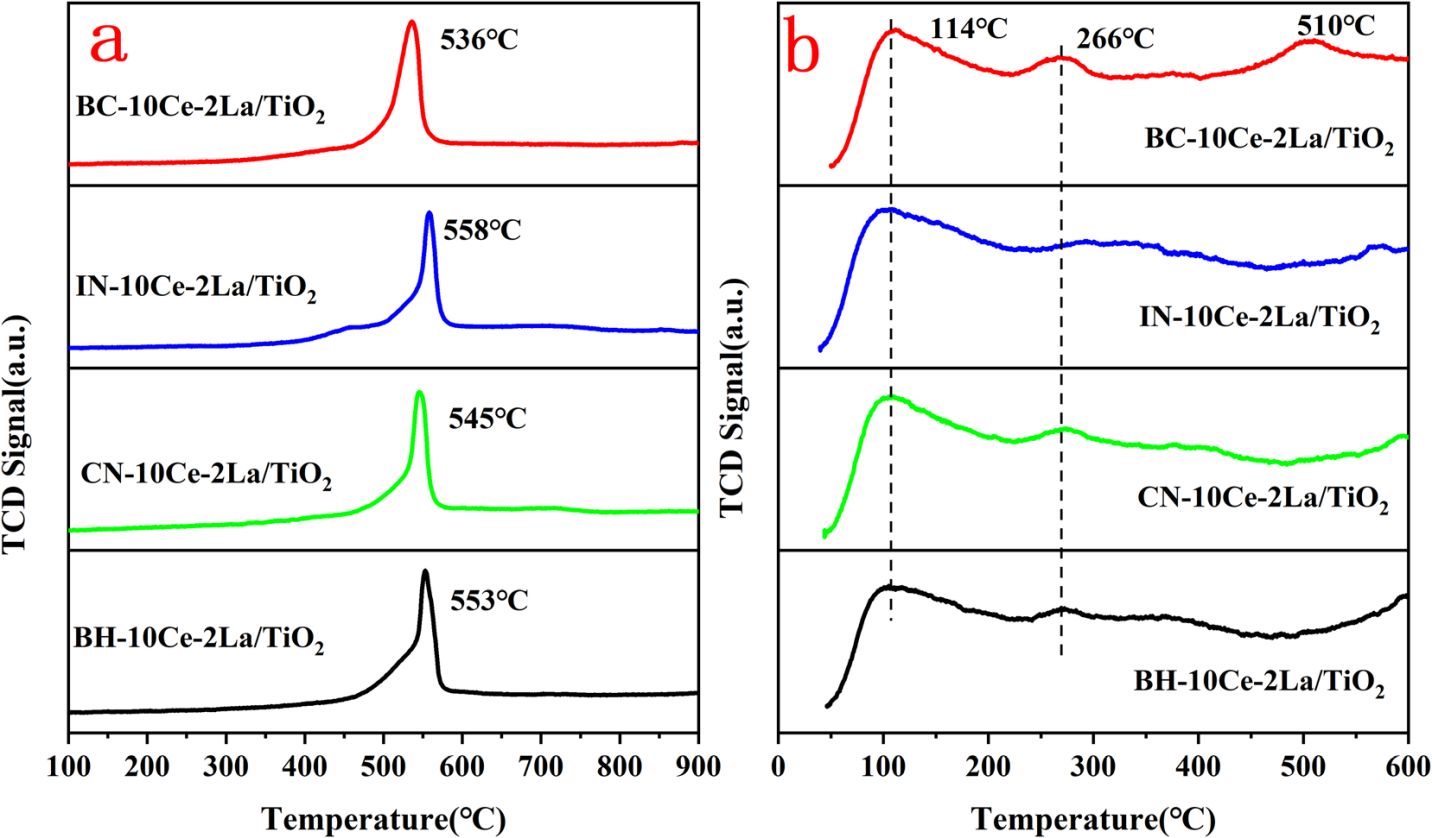
(a) H_2_-TPR and (b) NH_3_-TPD curves of BH-10Ce–2La/TiO_2_, CN-10Ce–2La/TiO_2_, IN-10Ce–2La/TiO_2_, and BC-10Ce–2La/TiO_2_ catalysts.

The redox properties of the catalysts were studied using H_2_-TPR and the results were shown in Fig. 4(a). All samples have reduction peaks at ∼550 °C, which can be attributed to the reduction of surface-ligated unsaturated Ce^4+^ that plays an important role in the oxidation reaction.^33^ In contrast, the smaller reduction peak over 600 °C is the reduction of the bulk phase of Ce^4+^ species.^34^ The hydrogen consumption of the reduction peak between 400 and 600 °C was quantified using CuO as the standard and listed in Table 4. Compared with other three samples, the reduction peak position of BC-10Ce–2La/TiO_2_ catalyst was the lowest and the hydrogen consumption (1.37 mmol g^−1^) was the highest, indicating the strongest redox ability. The results show that the surface active species of the BC-10Ce–2La/TiO_2_ are highly dispersed, which not only improves the redox capacity of the catalyst, but also enhances its oxygen storage capacity. The result is consistent with the XPS calculation.

**Table 4:** H_2_-TPR peak position and H_2_ consumption of the catalysts

Sample	Peak position (°C)	H_2_ consumption (mmol g^−1^)
BC-10Ce–2La/TiO_2_	536	1.37
IN-10Ce–2La/TiO_2_	558	1.21
CN-10Ce–2La/TiO_2_	545	1.16
BH-10Ce–2La/TiO_2_	553	0.93

The types and numbers of acidic sites of the catalysts were investigated by NH_3_-TPD and the results were shown in Fig. 4(b). The remarkable strong peaks below 200 °C and weak peaks at around 266 °C can be observed for all samples, which can be attributed to NH_3_ desorbed from the weak and medium to strong acid sites of the catalysts, respectively.^33^ For BC-10Ce–2La/TiO_2_, there is also a broad desorption peak at 510 °C which can be attributed to the desorption of ammonia ligated to strong acidic sites.^35^ Compared with the other three catalysts, the desorption peak of BC-10Ce–2La/TiO_2_ shifts toward higher temperatures and has a larger desorption peak area, implying that the most NH_3_ adsorbed on the surface.^36^ The above results show that the ball milling process can improve the quantity and the concentration of the acid sites on the catalyst, which helps to improve the denitrification efficiency of the catalyst.

### 3.2 Catalytic properties

#### 3.2.1. Activity of catalysts

**Fig. 5:**
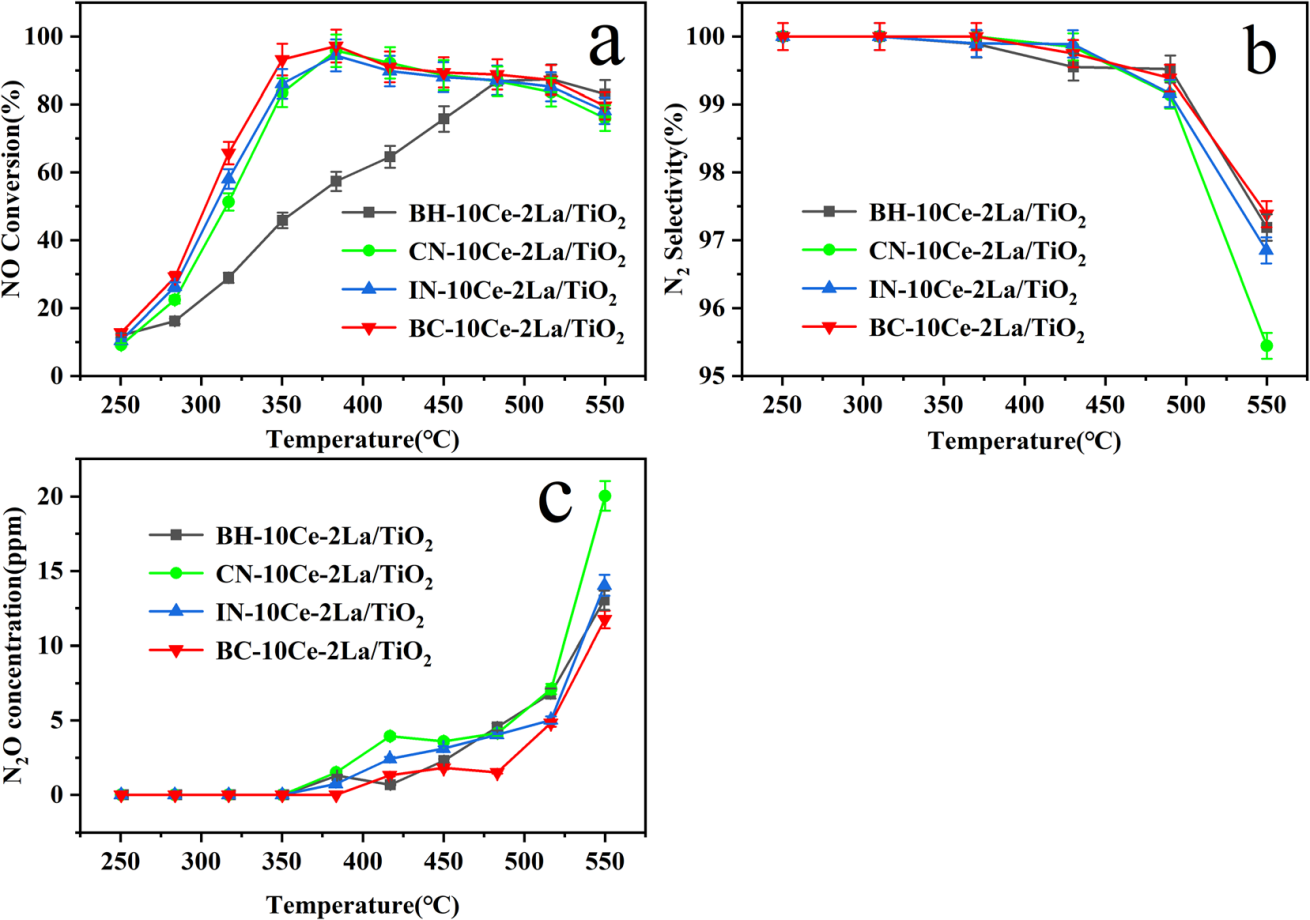
(a) NO conversion, (b) N_2_ selectivity, and (c) N_2_O concentration of BH-10Ce–2La/TiO_2_, CN-10Ce–2La/TiO_2_, IN-10Ce–2La/TiO_2_ and BC-10Ce–2La/TiO_2_ catalysts.

Fig. 5 shows the catalytic performance of 10Ce–2La/TiO_2_ catalysts prepared by different methods. As shown in Fig. 5(a), for BH-10Ce–2La/TiO_2_, with the increasing of the temperature, the NO conversion showed a slowly increasing trend from 250 to 500 °C, and then slowly decreased after reaching the highest NO conversion of 87.8% at 500 °C. The NO conversion of BC-10Ce–2La/TiO_2_ was greater than 80% in the temperature range of 330–550 °C and reached a maximum of 97.2% at 380 °C, which was slightly higher than those of IN-10Ce–2La/TiO_2_ and CN-10Ce–2La/TiO_2_, but much higher than that of BH-10Ce–2La/TiO_2_.

In addition to catalytic activity, N_2_ selectivity is also an important factor to evaluate the catalyst performance. As shown in Fig. 5(b) and (c), when the reaction temperature was higher than 350 °C, N_2_O started to generate, leading to a slow decrease of the N_2_ selectivity. In the reaction temperature interval, the N_2_ selectivity of all catalysts except CN-10Ce–2La/TiO_2_ is higher than 95.4%, which is very favorable for their practical applications. Considering that the preparation process of ball milling method has minimal environmental pollution and the products have the highest catalytic activity, BC-10Ce–2La/TiO_2_ should have a broad application prospect.

#### 3.2.2. Kinetic of reaction in the presence of catalysts

**Fig. 6:**
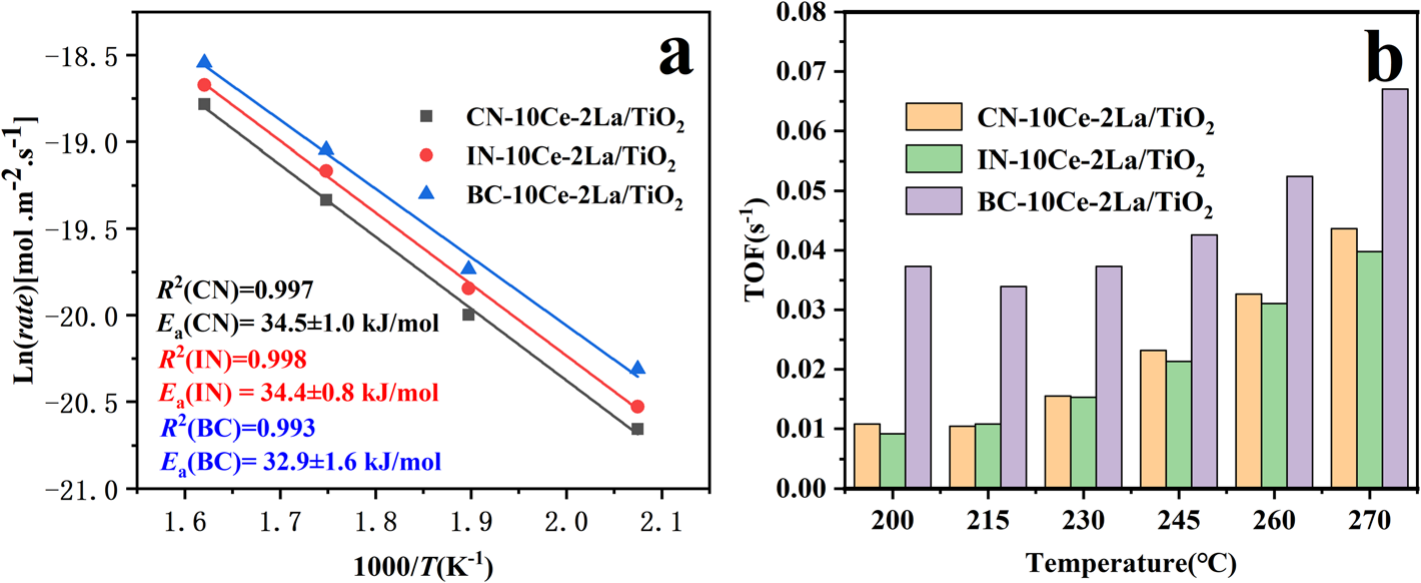
(a) Arrhenius plot in 250–350 °C, (b) TOF values in 200–270 °C of CN-10Ce–2La/TiO_2_, IN-10Ce–2La/TiO_2_ and BC-10Ce–2La/TiO_2_ catalysts.

For a catalytic reaction, activation energy is usually considered as a key factor to measure whether the reaction is easy to occur or not.^19^ The reaction rate of NO conversion per square meter of the catalysts in the temperature window of 250–350 °C was calculated according to eqn (3). By plotting Ln (*rate*) against 1/*T*, the activation energy of SCR reaction performance is determined *via* the slope of the line. Fig. 6 shows the Arrhenius plot in 250–350 °C and the TOF values in 200–270 °C of the CN-10Ce–2La/TiO_2_, IN-10Ce–2La/TiO_2_ and BC-10Ce–2La/TiO_2_ catalysts. As shown in Fig. 6(a), the activation energy of BC-10Ce–2La/TiO_2_ is 32.9 kJ mol^−1^, which is slightly lower than that of CN-10Ce–2La/TiO_2_ (34.5 kJ mol^−1^) and IN-10Ce–2La/TiO_2_ (34.4 kJ mol^−1^). The reason might be that for catalysts prepared by different methods, not all surface cerium ions are involved as active sites for NO_*x*_ reduction.^37^ The lower activation energy of BC-10Ce–2La/TiO_2_ also indicates that this catalyst requires less energy and is more likely to undergo catalytic reactions.^16,17^ To further understand the catalytic rate of different catalysts, the TOF value per Ce atom was calculated in the range of 200–270 °C. In the experiments, the maximum conversion of NO_*x*_ is controlled below 20% in the whole temperature range to eliminate the influence of diffusion. It is clear that the BC-10Ce–2La/TiO_2_ catalyst exhibits higher TOF values at each temperature than the other two catalysts, indicating that the BC-10Ce–2La/TiO_2_ has greater catalytic efficiency and higher intrinsic activity. According to the previous results, the reason may be owing to the synergistic effect between Ce and La catalysts, as well as more acidic sites and strength on the surface.^18,38^

### 3.3 Effect of preparation methods on the activity of Ce–La/TiO_2_ catalysts

Compared with impregnation method and co-precipitation method, the ball milling prepared catalyst shows excellent Ce^3+^ and O_α_ Ratio, which not only benefits from the carbonate raw materials, making the active components on the catalyst surface more dispersed, but also benefits from the physical treatment process of ball milling. The high-energy ball milling process can not only enhance the interaction between Ce oxide and other oxides, but also increase the fragmentation, extrusion and structural defects of the sample, which induces the transformation of Ce^4+^ to Ce^3+^ to a certain extent. Uniform active components will further improve the redox ability of the catalyst and improve the number and concentration of acid sites of the catalyst. Obviously, these advantages make BC-10Ce–2La/TiO_2_ have better catalytic performance and higher TOF value than other catalysts.

## 4. Conclusion

In conclusion, different preparation methods and precursors have an impact on the catalytic activity of high temperature catalysts. Compared with the catalysts prepared by conventional impregnation and co-precipitation methods, the ball milling method using carbonate instead of nitrate as raw material has uniform active sites, optimal redox properties and good acidic sites. Carbonate not only can induce CeO_2_ crystallization to a certain extent, but also avoid the secondary pollution problem caused by the release of NO_*x*_ during calcination with nitrate as raw material. In addition, the ball milling process has the advantages of simple operation, low grinding cost, no dust flying, and sustainable intermittent production. The less use of chemicals in the preparation process makes the environmental burden of the method relatively small, which is more in line with the requirements of green production and sustainable development, and provides a new idea for the preparation and production of denitrification catalysts.

## Author contributions

**Na Wang**: conceptualization, data curation, formal analysis, funding acquisition, investigation, methodology, project administration, supervision, validation, writing – review & editing. **Lei Wang**: writing – original draft, data curation, formal analysis, investigation, methodology. **Huidong Xie**: investigation, methodology, project administration, supervision. **Yang Liu**: investigation, methodology. **Yepeng Sun**: data curation, formal analysis. **Chang Yang**: data curation, formal analysis. **Chengmin Ge**: conceptualization, funding acquisition, supervision, validation.

## Conflicts of interest

The authors declare that they have no known competing financial interests or personal relationships that could have appeared to influence the work reported in this paper.

## Supplementary Material
